# Lac Felinum

**Published:** 1887-04

**Authors:** E. W. Berridge

**Affiliations:** London


### LAC FELINUM.

E. W. Berridge, M. D., London.

Mr. H., aged thirty-seven, September 10th, 1885, left eye inflamed for three weeks. Entire conjunctives bulbi a deep red. Photophobia. On left segment of left cornea is an ulcer. For last three nights, pain like a knife running from left eye to left occiput on lying down, especially when lying on left side, burning in left temple near eye, worse at night; has had to remain at home for the last five days. *Lac felinum*$^{40m}$ (Fincke) every four hours.

September 26th, says the eye was better next day, and continued to steadily improve. The pain ceased first, being entirely gone in three days; then the inflammation disappeared; now the left eye feels quite well, and there is only a slight film (of old standing) where the ulcer was.

Aggravation of eye pains by lying on left side has been verified by me in another published cure by *Lac felinum*. Aggravation of eye by lying on the painful side belongs to *Syphilinum*, and aggravation by lying on the unpainful side is under *Zincum*.

